# Nano-Iron as a Catalyst in Isocyanate-Free Rocket Propellants

**DOI:** 10.3390/polym17223006

**Published:** 2025-11-12

**Authors:** Michał Chmielarek, Beata Szczęśniak, Kamil Blacharski

**Affiliations:** High Energetic Materials Division, Faculty of Chemistry, Warsaw University of Technology, Noakowskiego 3, 00-664 Warsaw, Poland

**Keywords:** solid heterogeneous rocket propellant, isocyanate-free rocket propellant cross-linking system, combustion rate catalysts

## Abstract

This study investigates the influence of selected combustion rate catalysts on the ballistic, physicochemical, and mechanical properties of non-isocyanate heterogeneous solid rocket propellants. Methods for curing prepolymers and modifying hydroxyl-terminated polybutadiene (HTPB) to obtain carboxyl-terminated polybutadiene (CTPB) and its epoxidized derivative (EHTPB) are discussed. The initial stage involved the synthesis of CTPB and EHTPB. The obtained compounds were analyzed for viscosity, comparing their properties to those of the base polymer HTPB. FTIR spectra of the synthesized compounds were recorded. Crosslinking systems were formulated based on the synthesized substances and tested for tensile strength. The final stage consisted of preparing solid heterogeneous rocket propellants containing selected catalysts—catocene and iron nanopowder—and evaluating their burning rate, hardness, and density. The results of the rocket propellant tests indicate that both catalysts perform effectively in the proposed system. Significantly higher burning rates were achieved compared to the catalyst-free formulation. The addition of 1% catocene resulted in a 2.5-fold increase in burning rate. Even better performance was observed with iron nanopowder—1% addition led to an almost threefold increase in burning rate. Neither catalyst significantly affected the hardness of the propellant; all samples exhibited hardness values in the range of 71–76 Shore A. Increasing the catocene content led to a decrease in the final propellant density, whereas the addition of iron nanopowder increased the density relative to the base formulation.

## 1. Introduction

Solid heterogeneous rocket propellants are primarily used in rocket engines, which feature a simpler design compared to liquid-fueled engines. These propellants form the basis for the operation of anti-tank guided missiles, artillery rockets, and short-, medium-, and long-range surface-to-air missiles [1]. They are also present in auxiliary rocket engines of space shuttles and launch vehicles. Rocket propellants composed of hydroxyl-terminated polybutadiene (HTPB), ammonium perchlorate (AP), and aluminum (Al) are widely used due to their low cost, favorable burning rate, and good mechanical properties [2,3].

Modifications to solid rocket propellants aim not only to improve their ballistic and mechanical properties but also to enhance performance while reducing production costs. An important aspect of current research is minimizing the environmental impact of these materials. To this end, studies are conducted to develop environmentally friendly propellants with properties comparable to or better than those of currently used formulations.

Heterogeneous rocket propellant is a non-uniform mixture in which solid particles are embedded within a polymer matrix. Upon crosslinking at elevated temperatures, the system forms a compact mass with defined physicomechanical properties. The primary components include binders—which simultaneously serve as fuel—and oxidizers. Additionally, metallic fuels and technological additives such as stabilizers, antioxidants, and burn rate modifiers are commonly used [4].

A catalyst is a compound that can influence the rate of a chemical reaction by providing an alternative reaction pathway with a lower energy profile. It affects only the reaction rate. Despite its typically low mass fraction in the mixture (usually not exceeding a few percent), a catalyst can significantly impact the processing behavior of the mixture as well as the physicomechanical properties of the final product. It is crucial that the catalyst fulfills its role while remaining compatible with other components and not adversely affecting the structure or stability of the rocket propellant [5,6].

Nanoscience has recently become a major focus of research, and the trend toward miniaturization has also extended to catalytic processes. Materials reduced to the nanoscale may exhibit novel properties, thereby expanding their potential applications. Catalytic activity is primarily a surface phenomenon, and reducing the size of the catalyst increases its specific surface area, which in turn enhances its catalytic activity.

Nanometals are more effective catalysts than metal oxide nanoparticles. During the decomposition process, the metal reacts with oxygen molecules generated in situ. The formation of metal oxides is accompanied by significant heat release, which gives nanometals an advantage over metal oxide nanoparticles in terms of catalytic activity. The presence of copper (Cu), nickel (Ni), and aluminum (Al) nanopowders in propellant formulations lowers the activation energy of the high-temperature decomposition of ammonium perchlorate (AP). The same applies to the use of bimetallic nanalloys such as Ni-Co, Ni-Cu, and Ni-Zn [7,8]. A further group of catalysts includes nanoparticles of mixed transition metal oxides (MTMO), which include MnO_2_ and Cr_2_O_3_ [9,10,11]. A popular group of catalysts are compounds containing ferrocene, which are primarily used in small-caliber and short-range rockets [12,13]. Nanomaterials are not only used as rocket propellants. They are also used in many technologies in the rocket production process. They are used in everything from the coating protecting the rocket propellant grain or the engine itself to the main structure of the missile [14,15].

The crosslinking agent plays a crucial role in determining the mechanical properties of composite solid rocket propellants [16,17].

The preferred mechanism for this process is polyaddition. In contrast, elimination reactions produce volatile by-products that can lead to cracks or voids in the cured material. The crosslinking process should exhibit sufficiently slow curing kinetics to allow for casting of the mixture; the system must maintain adequate pot life. Additionally, the curing temperature should not be excessively high to avoid damaging the final product’s casing.

The result of crosslinking is the formation of three-dimensional bonds between prepolymer chains and the crosslinking system. The number of these bonds—i.e., the degree of crosslinking—has a significant impact on the mechanical properties of the heterogeneous rocket propellant [17,18].

**Figure 1:**
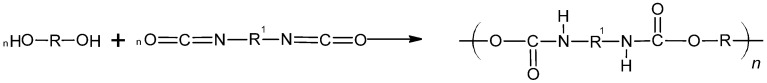
Scheme for obtaining polyurethanes.

Isocyanates are commonly used as crosslinking agents. They form polyurethane bonds with binder molecules during the curing process. Such systems exhibit excellent binding properties. The reaction proceeds according to the following scheme (Figure 1).

Isocyanate-based rocket propellants exhibit several disadvantages. During decomposition, they release toxic hydrogen cyanide (HCN), posing health risks to users. Additionally, these propellants are highly sensitive to moisture present in their components, which can lead to the formation of voids in the cured material. This reduces the charge density, alters mechanical strength, and affects the burning rate characteristics [19,20,21].

Isocyanate propellants are incompatible with emerging rocket propellant components such as iron nanopowder and ammonium dinitramide (ADN). Until now, the most commonly used catalysts have been iron oxide and ferrocene derivatives, with AP serving as the oxidizer. However, modern weapon systems require modified propellant formulations and the incorporation of new ingredients.

In this study, a novel catalyst was used in combination with the conventional oxidizer AP. Therefore, an alternative polymeric crosslinking system based on HTPB derivatives was employed.

## 2. Materials and Methods

**Figure 2:**
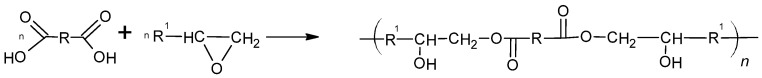
Scheme of the reaction of CTPB with an epoxy group.

The crosslinking system used in the prepared propellants consists of CTPB (as the binder) and EHTPB (as the curing agent). The EHTPB employed contains approximately 30% epoxidized double bonds, which enables it to act as a crosslinker and effectively cure the final propellant mixture. Both components of the system were synthesized according to procedures available in the literature [22,23,24,25]. The reaction scheme for the curing process in the described system is presented below (Figure 2).

Prior to the preparation of rocket propellants, the properties of the prepolymers and crosslinking agents as well as the crosslinking systems based on them were characterized. Studies were conducted on three polymers: HTPB, commercially known as Poly bd R45 HTLO; CTPB and EHTPB. The basic characteristics of the rocket propellants obtained are presented below in the form of density, FTIR and hardness tests.

In addition to density, viscosity is one of the most critical thermophysical parameters for characterizing the technical properties of fluids. Unfavorable rheological properties can lead to cracks and voids in rocket propellants. Structural defects in the propellant may result in detonation due to a rapid increase in the burning surface area. Therefore, proper selection of the mixture’s viscosity is essential [26].

The viscosity of the prepolymers and crosslinking agents used is a key factor in selecting appropriate components. Low viscosity at the mixing temperature (typically 65 °C) allows for effective blending of ingredients and facilitates the incorporation of a higher proportion of solid components, such as oxidizers and metallic fuels. The viscosity of the mixture influences the curing and crosslinking processes within the internal structure, as well as its pot life [27].

During the casting process, it is crucial to maintain the time window in which the mixture can be poured properly to ensure a homogeneous consistency. If the viscosity of the mixture changes significantly over time, the quality of the resulting propellant grain may vary depending on when it was cast—either at the beginning or end of the process [28].

The density of solid rocket propellants is a critical parameter due to its impact on combustion chamber design and burning rate performance. A high charge density is desirable, as it allows for the use of a smaller combustion chamber, directly contributing to reduced engine production costs. Low density may indicate the presence of voids in the cured material. Such structural defects are highly undesirable, as they not only reduce density but also alter the mechanical and ballistic properties of the propellant grain.

### 2.1. Raw Materials

CTPB—Synthesized in the laboratory from HTPB R45 HTLO (Cray Valley, Saint-Avold, France). EHTPB—Epoxidized derivative containing approximately 30% epoxidized double bonds, synthesized in the laboratory of High energetic Materials Division, Faculty of Chemistry, Warsaw University of Technology from HTPB R45 HTLO (Cray Valley). AP—Supplied by Eruca Technologies s.r.o., Bohumín, Czech Republic, Dioctyl adipate (DOA)—Purity > 98%, purchased from Merck, Warsaw, Poland. Catocene—Purity > 98%, obtained from Neo Organics, Otwock, Poland. Iron nanopowder (Nano Fe)—Purity 99.9%, particle size 40–60 nm, supplied by Pol-Aura, Morąg, Poland.

### 2.2. Method of Obtaining of Rocket Propellant

Propellant mixing was carried out using a NETZSCH Germany PML1 planetary mixer. The mixer has a 1 L capacity, is equipped with a planetary stirrer, and is connected to a vacuum pump. Prior to adding the ingredients, the mixing vessel was thermostated to 65 °C. First, CTPB, ADO, and AO 2246 were added to the reactor. The mixer was sealed, the mechanical stirrer activated, and the contents mixed for 5 min. Then, the vacuum pump was started and mixing continued for another 10 min. After this, the system was vented, the stirrer turned off, and the mixer opened. A fine-particle fraction of AP was added. The mixer was sealed again, the stirrer activated, and mixing continued for 20 min. Afterward, the mixer was opened and the first portion of coarse-particle AP was added. The mixer was resealed, the stirrer restarted, and mixing continued for another 20 min. The same procedure was repeated for the second portion of coarse AP. After 20 min of mixing, the stirrer was turned off, the mixer opened, and the catalyst was added. The mixer was sealed, the stirrer activated, and mixing continued for 5 min. Then, the vacuum pump was turned on and mixing continued for an additional 45 min. Next, the system was vented, the stirrer turned off, and the mixer opened to add the crosslinking agent. The mixer was sealed again, the stirrer activated, and the contents mixed for 5 min. The vacuum pump was then turned on and mixing continued for 10 min. Finally, the system was vented, the stirrer turned off, and the mixer opened.

Seven propellant mixtures were prepared (Table 1). Propellant 1 served as the base formulation, without any catalyst additives. Propellants 2, 3, and 4 contained 1%, 2%, and 3% of the catalyst in the form of catocene, respectively. Propellants 5, 6, and 7 were formulated with 0.5%, 0.75%, and 1% of the catalyst in the form of iron nanopowder, respectively.

### 2.3. Methods of Analysis

Fourier-transform infrared (FTIR) spectroscopy is an analytical technique used to investigate the molecular structure and composition of compound mixtures. FTIR measurements were performed using a Nicolet 6700 FTIR spectrometer (Thermo Scientific, Warsaw, Poland) with compatible OMNIC 8.1.0.10 software. Prior to sample analysis, a background scan was conducted. A drop of the test compound was placed on the measurement plate, and the ATR accessory was secured. The measurement was then carried out. Between successive measurements, the plate and ATR crystal were cleaned using a lint-free wipe moistened with technical-grade acetone.

The static tensile test is a fundamental and widely used method for evaluating material strength. This test enables the determination of tensile strength, breaking stress, and the distinct or conventional yield point. The experiment was conducted using an Instron 3366 (Instron Poland, Opole, Poland) universal testing machine equipped with Bluehill 3 software. Samples were prepared by placing appropriate amounts of prepolymer and crosslinking agent into silicone molds, followed by vigorous mixing with a wooden spatula to ensure homogeneity. The prepared samples were cured for 24 h in a laboratory oven at 65 °C. After curing, the silicone molds were removed, and rectangular specimens of appropriate dimensions were cut using a scalpel. Specimens with a thickness of 2.5 mm, length of 65 mm, and width ranging from 4.5 to 5.7 mm were placed in the grips of the testing machine. The test involved axial stretching of the sample in opposite directions at room temperature, continued until the specimen ruptured.

Density measurements were performed using a non-destructive helium pycnometer, AccuPyc II 1340 (Micromeritics Poland, Poniszowice, Poland). The device calculates the density of the tested material based on the measured volume of the propellant and the known mass of the sample entered into the instrument’s operating software.

Hardness of the propellant provides insight into the degree of material curing. Abnormally low hardness values may indicate uncontrolled aging of the propellant, associated with plasticizer loss through diffusion and oxidation of the polymer matrix. Significant variation in hardness measurements across a single sample may suggest surface heterogeneity caused by component migration within the grain or chemical interactions between individual ingredients [28].

Hardness testing was conducted using a Shore-type analog durometer, HBA 100-0 (CAL NARZĘDZIA, Kraków, Poland). The prepared propellant sample was placed on a flat surface. The durometer was applied perpendicularly to the material and pressed smoothly for approximately 2 s. The deflection of the passive needle was used to read the measurement result.

The burning rate of composite propellants is considered one of the most influential parameters governing the ballistic performance of solid rocket motors. Accurate knowledge of burning rate is essential for determining the operational characteristics of the engine. Combustion chamber pressure has a significant effect on burning rate. The burning behavior is primarily influenced by the properties of oxidizer particles, burn rate catalysts, and the addition of metallic fuel [17,29,30].

Propellant samples were prepared in the form of uninhibited cylindrical grains for each of the produced propellant formulations. Combustion tests were carried out using a laboratory-scale rocket motor consisting of a nozzle section with interchangeable nozzles, a cylindrical combustion chamber, the propellant grain, an igniter, and a chamber closure. The system was equipped with a pressure sensor, amplifier, and a computer system with software for monitoring and recording combustion time characteristics and chamber pressure.

Nozzles with diameters ranging from 2.2 mm to 3.5 mm were installed in the setup. A cylindrical propellant grain was then placed inside the combustion chamber. The igniter was constructed from an initiating charge composed of a 0.2 A electric igniter, a black powder pellet measuring 6 × 2 mm and weighing 0.2 g. The igniter was positioned behind the propellant grain inside the chamber.

Combustion was initiated, and the chamber pressure was recorded throughout the burning process. This allowed for the determination of the maximum chamber pressure during combustion and the burning rate of the propellant.

## 3. Results and Discussion

### 3.1. Viscosity

**Figure 3:**
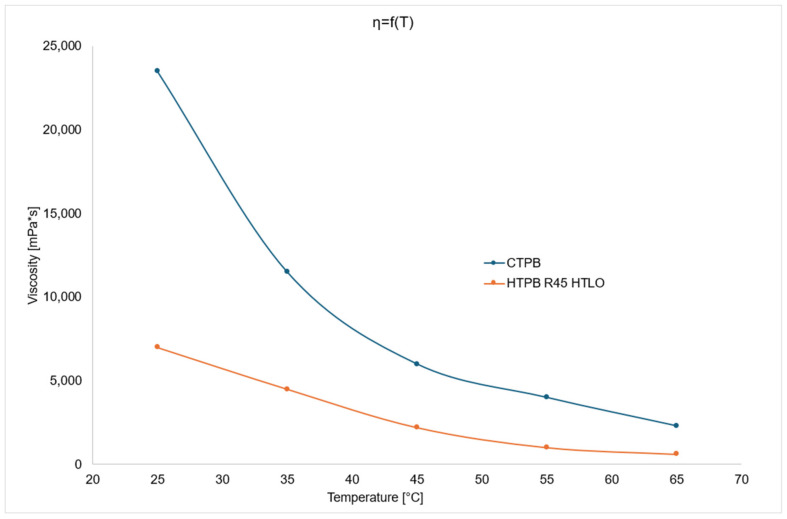
Viscosity change chart as a function of temperature for CTPB and HTPB.

The viscosity changes of HTPB R45 HTLO and CTPB polymers as a function of temperature are shown in the figure below (Figure 3).

The above graph comparing viscosity changes as a function of temperature for both polymers reveals a significant difference in viscosity values at 25 °C. As the temperature increases, this difference gradually decreases. At 65 °C—the most critical temperature within the examined range, as it is commonly used for propellant mixing—the viscosity of HTPB R45 HTLO is 912 mPa·s, while that of CTPB is 2218 mPa·s.

This analysis demonstrates that both compounds can be effectively used as prepolymers in binders for solid heterogeneous rocket propellants. The lower viscosity of HTPB R45 HTLO compared to CTPB allows for the incorporation of a greater amount of solid components and improves miscibility with other propellant ingredients.

**Figure 4:**
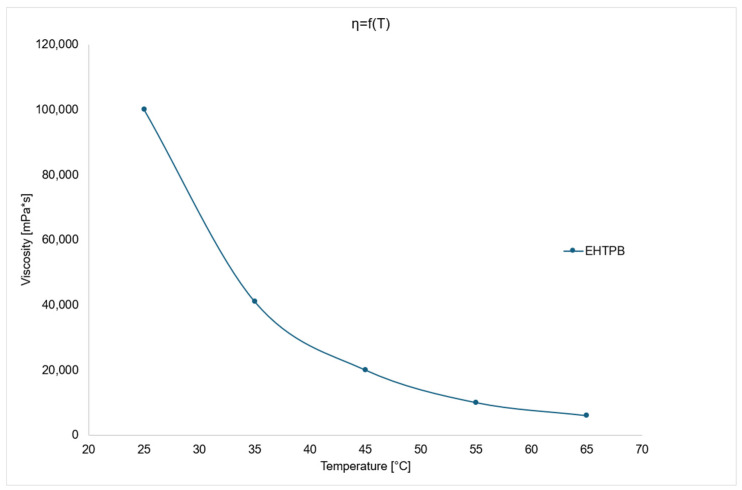
Viscosity change chart as a function of temperature for EHTPB.

The Figure 4 above shows that EHTPB has high viscosity at both 25 and 65 °C. Such high viscosity is caused by the high molecular weight of the compound and the significant degree of epoxidation of HTPB double bonds.

### 3.2. FTIR

**Figure 5:**
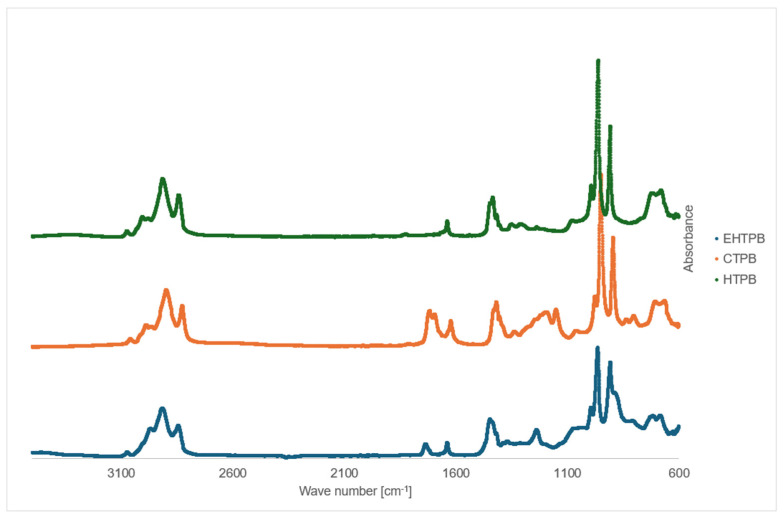
FTIR spectrum of HTPB, CTPB and EHTPB.

The FTIR spectrum (Figure 5) obtained for HTPB shows absorption bands in the range of 3100–2800 cm^−1^, corresponding to the stretching vibrations of hydroxyl groups, as well as a band at 1640 cm^−1^ associated with the stretching vibrations of C=C double bonds. When comparing the spectra of both prepolymers, the FTIR spectrum of CTPB reveals distinct bands attributed to the stretching vibrations of C=O bonds in the range of 1780–1650 cm^−1^, along with a peak corresponding to C–O vibrations between 1300–1150 cm^−1^. This indicates the presence of ester and carboxyl groups in the analyzed compound, confirming the successful synthesis of CTPB.

The FTIR spectrum of the crosslinking agent EHTPB, similar to the previously discussed compounds, shows an intense band in the region of 3000–2800 cm^−1^ corresponding to the stretching vibrations of hydroxyl (OH) groups. In the range of 1750–1650 cm^−1^, bands associated with carbonyl (C=O) groups are observed, while the region between 1450–1200 cm^−1^ displays bands indicative of C–O bonds in the synthesized compound. These bands appear with high intensity, confirming the successful synthesis of the desired product.

### 3.3. Mechanical Properties

Test samples were prepared using silicone molds. A calculated amount of CTPB prepolymer and EHTPB crosslinking agent was added to each mold to ensure the sample mass ranged between 4.5 and 5 g. The contents were then vigorously mixed using a wooden spatula. The samples were placed in a laboratory oven for 24 h at 65 °C. After curing, the resulting systems were evaluated visually.

Visual analysis of the cured systems indicated that optimal crosslinker concentrations in the CTPB-based formulation are 15% and 20%. Based on the conducted study, it was concluded that a 15% concentration of the crosslinking agent is sufficient to produce a propellant mixture with good mechanical properties. In contrast, using 10% EHTPB did not yield a cured product.

For each sample, the following mechanical parameters were measured: Tensile strength (Rm), Maximum tensile force (Fmax), Elongation at break (A). The table below presents the averaged results of these measurements (Table 2).

Analysis of the averaged values of the measured parameters indicates that increasing the concentration of the crosslinking agent in the mixture leads to higher elongation at break. A pronounced, nearly twofold increase in this parameter is observed when the EHTPB content is raised from 10% to 15%. However, the degree of curing does not affect the tensile strength or the maximum tensile force.

### 3.4. Properties of Manufactured Rocket Propellants

**Figure 6:**
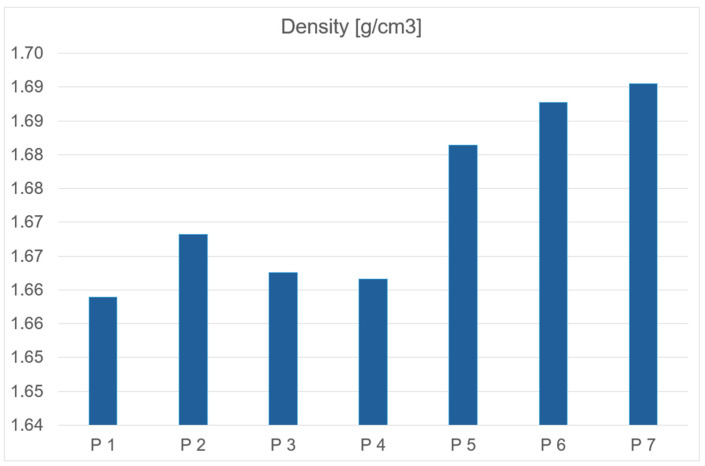
Dependency graph of propellants density.

All analyzed fuel samples exhibit comparable density values. Both propellants containing catocene and those with iron nanopowder additives demonstrate slightly higher densities relative to the base propellant. In the case of propellants with liquid catocene as a catalyst, density decreases with increasing catalyst concentration in the sample mass. Conversely, for fuels containing iron nanoparticle additives, density increases proportionally with the catalyst content in the sample. The density data is presented below in graph form to visualize the results (Figure 6 and Table 3).

The base propellant, without any catalytic additives, exhibits a hardness of 74 Shore A. The introduction of a liquid catalyst, specifically catocene, results in a slight reduction in hardness across subsequent samples. A progressive decrease in hardness is observed with increasing concentrations of the liquid catalyst, as demonstrated in propellant samples 2, 3, and 4.

**Figure 7:**
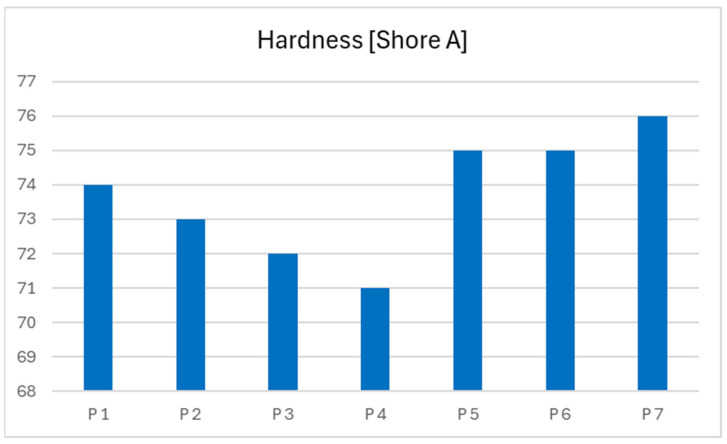
Dependency graph of propellants hardness.

In contrast, the addition of a solid catalyst in the form of iron nanopowder leads to a modest increase in the hardness of the resulting propellant formulations. This effect is consistent with the expected reinforcement behavior of solid particulate additives. The data is presented below in graph form Table 4 to visualize the results (Figure 7).

Nevertheless, all modified propellant samples maintain a satisfactory level of hardness. No significant impact of the applied burn rate modifiers on the crosslinking behavior or mechanical integrity of the cured propellant matrix has been observed.

Each of the prepared propellant samples containing catalytic additives exhibits a significantly higher burn rate compared to the base formulation. This confirms that the selected modifiers are well-suited for the given system.

These results are particularly promising in the case of the nano-Fe catalyst, which had previously been excluded from use due to its incompatibility with isocyanate-based systems. The burn rate increases proportionally with the catalyst concentration in the mixture, both for propellants containing catocene and those incorporating iron nanopowder.

**Figure 8:**
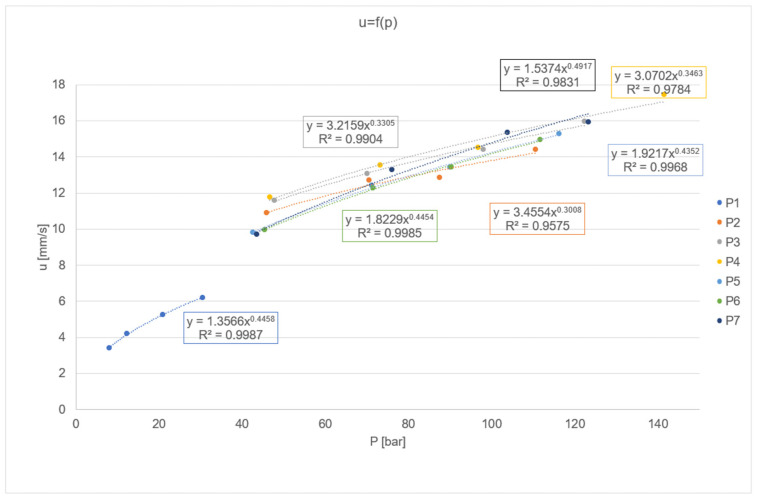
Dependency graph of burning rate on maximum pressure.

The highest burn rate was observed in the sample containing the greatest amount of catalyst—specifically, 3% catocene by mass. When comparing samples with identical modifier concentrations, namely 1% catocene versus 1% iron nanopowder, the propellant with nano-Fe demonstrates a superior burn rate (approximately 16 mm/s versus 14 mm/s for the catocene-based formulation). The data is presented below on Figure 8.

## 4. Conclusions

The aim of the presented study was to investigate the influence of selected catalysts on the properties of non-isocyanate heterogeneous solid rocket propellants. The evaluation of individual components and developed crosslinking systems enabled the selection of a formulation with optimal performance characteristics. Based on the obtained results, heterogeneous propellant mixtures were prepared with varying concentrations of the selected catalysts. The final propellants were analyzed in terms of density, hardness, and burn rate.

Both applied catalysts effectively enhance the burn rate. For propellants containing either catocene or iron nanopowder, the burn rate increases with higher catalyst concentrations. A comparison of samples with identical catalyst content indicates superior ballistic performance for iron nanopowder.

All prepared propellant samples meet the required hardness specifications. No significant influence of the catalysts on this parameter was observed.

The addition of catocene results in a decrease in propellant density, which becomes more pronounced with increasing catalyst concentration. In contrast, the inclusion of iron nanopowder increases the density of the final product, with values rising proportionally to the modifier content.

The present study has demonstrated that a non-isocyanate binder system composed of two liquid polymers can be successfully employed in solid rocket propellant formulations, both with the well-established catalyst catocene and with nano-iron, which had not previously been used due to compatibility concerns.

## Figures and Tables

**Table 1:** Composition of obtained rocket propellants.

Component [%]	Rocket Propellant Sample Number						
	1	2	3	4	5	6	7
CTPB/EHTPB	12.5	12.5	12.5	12.5	12.5	12.5	12.5
ADO	2.5	2.5	2.5	2.5	2.5	2.5	2.5
Antioxidant AO 2246	0.2	0.2	0.2	0.2	0.2	0.2	0.2
AP	84.8	83.8	82.8	81.8	84.3	84.05	83.8
Catocene	-	1	2	3	-	-	-
Nano Fe	-	-	-	-	0.5	0.75	1

**Table 2:** Summary of average values obtained for CTPB-EHTPB systems.

EHTPB Cross-Linking Agent Content [%]	Rm [Mpa]	Fmax [N]	A [%]
10	0.23	3.19	8.96
15	0.41	5.52	17.04
20	0.28	3.43	20.92

**Table 3:** Density of obtained rocket propellants.

Sample Number	Density [g/cm^3^]
P 1	1.6589
P 2	1.6682
P 3	1.6626
P 4	1.6616
P 5	1.6814
P 6	1.6877
P 7	1.6905

**Table 4:** Hardness of obtained rocket propellants.

Sample Number	Hardness [Shore A]
P 1	74
P 2	73
P 3	72
P 4	71
P 5	75
P 6	75
P 7	76

## Data Availability

The original contributions presented in this study are included in the article. Further inquiries can be directed to the corresponding author.
